# EXPOSURE SCIENCE: BPA in Canadian Population Highest Among Teens

**DOI:** 10.1289/ehp.118-a430a

**Published:** 2010-10

**Authors:** Julia R. Barrett

**Affiliations:** **Julia R. Barrett**, MS, ELS, a Madison, WI–based science writer and editor, has written for *EHP* since 1996. She is a member of the National Association of Science Writers and the Board of Editors in the Life Sciences

For the first time, estimated bisphenol A (BPA) concentrations in the general Canadian population are available, and teenagers are leading the way in terms of exposure.^1^ Data collected during 2007–2009 show that 91% of Canadians tested had detectable levels of BPA in their urine, indicating widespread exposure to the chemical among the general population. Among age groups, teenagers (12–19 years old) had the highest geometric mean level, 1.50 μg/L, compared with the overall geometric mean for 6- to 79-year-olds of 1.16 μg/L.

The August 2010 report from Statistics Canada is based on the Canadian Health Measures Survey, an ongoing effort to collect biomonitoring data from a nationally representative sample—comparable to the U.S. National Health and Nutrition Examination Survey (NHANES). “A definitive answer as to why we observed higher concentrations among teenagers would be useful,” says Tracey Bushnik of Statistics Canada and lead author of the report. “Beyond making some general hypotheses, however, we can’t really speak to what may be driving these differences.”

BPA is a high-volume industrial chemical with many applications. Food and beverage packaging represents the largest source of human exposure due to the compound leaching into packaged contents from container linings.^2^ Animal studies of low-level BPA exposure suggest negative effects on the reproductive system and neurodevelopment, increased risks of prostate and mammary cancers, and possibly higher risk of obesity and diabetes.^2^–^5^ Although exposure levels have been established in humans, definitive data for related health effects are not available. Similarly, data establishing a link between BPA intake and biomonitoring results are also lacking.

A recent study based on 2005–2006 NHANES data took a preliminary look at potential sources of BPA exposure.^6^ This analysis supported an association between urinary BPA concentrations and consumption of soda, school lunches, and meals prepared outside the home, all of which likely involve packaged foods, including canned goods. Eventually other sources and routes of exposure may be identified as well, as suggested by the recently publicized case of BPA found on cash register receipts and thermal papers.^7^

Such findings need to be viewed with caution though. “These results are best considered as hypothesis-generating,” says Judy LaKind, president of LaKind Associates in Catonsville, Maryland, and lead author of the NHANES analysis. She adds, “Further research is needed—preferably research that includes actual measurements of BPA in [sources of exposure]—to substantiate these results.” LaKind stresses that the value of biomonitoring studies lies in providing reference ranges, trend data, and the bases for research hypotheses; they do not establish causal relationships.^8^

With the Canadian study in mind, Bushnik agrees that biomonitoring studies are important for creating a foundation for more in-depth study. “With these data we have baseline information against which we can compare future data,” she says. Once more data are available, it will be possible to examine BPA exposure in greater detail and possibly also consider interrelationships of personal variables and sources of exposure.
